# Increased spread and replication efficiency of *Listeria monocytogenes* in organotypic brain-slices is related to multilocus variable number of tandem repeat analysis (MLVA) complex

**DOI:** 10.1186/s12866-015-0454-0

**Published:** 2015-07-03

**Authors:** Claudia Guldimann, Michelle Bärtschi, Joachim Frey, Andreas Zurbriggen, Torsten Seuberlich, Anna Oevermann

**Affiliations:** Division of Neurological Sciences, Neurocenter, Department of Clinical Research and Veterinary Public Health, Vetsuisse Faculty, University of Bern, Bern, Switzerland; Graduate school for Cellular and Biomedical Sciences, University of Bern, Bern, Switzerland; Institute of Veterinary Bacteriology, Vetsuisse Faculty, University of Bern, Bern, Switzerland

**Keywords:** *Listeria monocytogenes*, Rhombencephalitis, Neurovirulence, Organotypic brain slice, Plaque test, *In vitro* model, Ruminant, Microglia, MLVA complex

## Abstract

**Background:**

*Listeria (L.) monocytogenes* causes fatal infections in many species including ruminants and humans. In ruminants, rhombencephalitis is the most prevalent form of listeriosis. Using multilocus variable number tandem repeat analysis (MLVA) we recently showed that *L. monocytogenes* isolates from ruminant rhombencephalitis cases are distributed over three genetic complexes (designated A, B and C). However, the majority of rhombencephalitis strains and virtually all those isolated from cattle cluster in MLVA complex A, indicating that strains of this complex may have increased neurotropism and neurovirulence. The aim of this study was to investigate whether ruminant rhombencephalitis strains have an increased ability to propagate in the bovine hippocampal brain-slice model and can be discriminated from strains of other sources. For this study, forty-seven strains were selected and assayed on brain-slice cultures, a bovine macrophage cell line (BoMac) and a human colorectal adenocarcinoma cell line (Caco-2). They were isolated from ruminant rhombencephalitis cases (*n* = 21) and other sources including the environment, food, human neurolisteriosis cases and ruminant/human non-encephalitic infection cases (*n* = 26).

**Results:**

All but one *L. monocytogenes* strain replicated in brain slices, irrespectively of the source of the isolate or MLVA complex. The replication of strains from MLVA complex A was increased in hippocampal brain-slice cultures compared to complex C. Immunofluorescence revealed that microglia are the main target cells for *L. monocytogenes* and that strains from MLVA complex A caused larger infection foci than strains from MLVA complex C. Additionally, they caused larger plaques in BoMac cells, but not CaCo-2 cells.

**Conclusions:**

Our brain slice model data shows that all *L. monocytogenes* strains should be considered potentially neurovirulent. Secondly, encephalitis strains cannot be conclusively discriminated from non-encephalitis strains with the bovine organotypic brain slice model. The data indicates that MLVA complex A strains are particularly adept at establishing encephalitis possibly by virtue of their higher resistance to antibacterial defense mechanisms in microglia cells, the main target of *L. monocytogenes.*

**Electronic supplementary material:**

The online version of this article (doi:10.1186/s12866-015-0454-0) contains supplementary material, which is available to authorized users.

## Background

The Gram + bacterium *Listeria* (*L.*) *monocytogenes* is an opportunistic food-borne pathogen with considerable impact on human and livestock health and food safety. It causes listeriosis [1,2], which may manifest in distinct clinical forms including febrile gastroenteritis, abortions, septicemia, and neurolisteriosis [2,3] and is associated with high mortality [4,5]. In humans, *L. monocytogenes* is commonly isolated in the context of meningitis [6], and neurolisteriosis is responsible for high fatality rates and chronic sequelae [7–10]. In farmed ruminants, neurolisteriosis is amongst the most common causes of central nervous system (CNS) disorders and characteristically presents as encephalitis, which targets the brainstem (rhombencephalitis) and is often deadly [11–14]. Clinical observations in livestock may indicate differences in organ tropism between *L. monocytogenes* strains. Different clinical forms of listeriosis rarely overlap in the same ruminant herd or in the same animal during an outbreak [15,16]. Rhombencephalitis generally occurs without involvement of other organs and without inducing abortion in pregnant ruminants [17–19].

The ubiquitous nature of *L. monocytogenes* as a saprophytic soil inhabitant constitutes a challenge for surveillance and effective disease control [20]. *L. monocytogenes* is divided into four phylogenetic lineages [21] as determined by various genotypic and phenotypic subtyping tools [22–27] and may differ in virulence and the potential to cause epidemic outbreaks [28–33]. For instance, of the two major phylogenetic lineages I and II, which are associated with human and animal infections, lineage I is overrepresented in clinical isolates [21,22,28,31]. In contrast, lineage II strains are more commonly isolated from food and the environment. The two minor lineages III and IV are rarely isolated and are associated with ruminant infections [21]. From the 13 known serotypes, serotype 4b (belonging to lineage I) is associated with most of the severe clinical cases and the majority of outbreaks [34,35]. Nonetheless, available subtyping methods cannot predict the virulence of a given isolate, and the propensity of certain subtypes to cause sporadic illness, epidemic outbreaks or specific clinical syndromes (*e.g.* neurolisteriosis) remains poorly understood [36,37].

There have been few systematic investigations of the genetic diversity of *L. monocytogenes* strains isolated from ruminants [31,38–40]. Using multilocus variable number tandem repeat analysis (MLVA) of 183 isolates, we could show that ruminant rhombencephalitis strains are found predominantly in MLVA complex A and also B, belonging to lineage I, and to a lesser extent to complex C of lineage II [31]. Nearly all rhombencephalitis strains from cattle cluster in MLVA complex A indicating that strains of this genetic complex may have increased neurotropism and neurovirulence [31]. This observation is in line with studies showing that lineage I strains are overrepresented in rhombencephalitis, whereas lineage II strains are equally associated with rhombencephalitis, septicemia and fetal infections [38,39].

The lack of a relevant model hinders experimental determination of neurovirulent potential and neurovirulence mechanisms of *L. monocytogenes* strains and means that neurovirulence can at present only be defined using data from neurolisteriosis cases [41]. In a previous study, we developed a bovine organotypic hippocampal brain-slice model, which is susceptible to *L. monocytogenes* [42]. The aim of the present study was to investigate if ruminant rhombencephalitis strains can be discriminated from non-encephalitic strains using the *in vitro* CNS model. To this end, bovine hippocampal slices were infected with a panel of 47 selected *L. monocytogenes* strains from various clinical, environmental and food sources. Replication and spread of strains within brain-slice cultures were analyzed by determination of CFU’s and size of infection foci. These results were correlated with the source of the isolate and MLVA–complex of the respective strains and compared to plaque test results in two cell lines.

## Results

### Infection of organotypic brain-slice cultures with *L. monocytogenes* strains

**Fig. 1:**
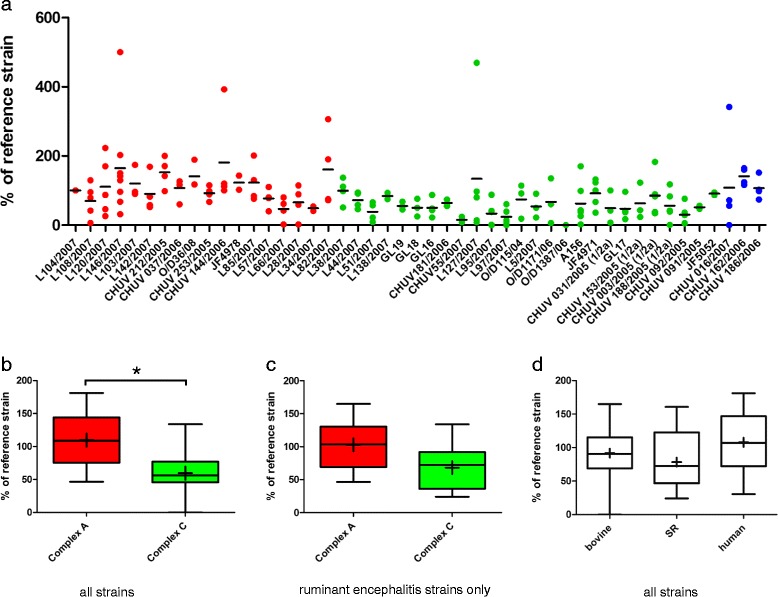
Replication of *L. monocytogenes* strains in brain-slices. Results are shown relative to the internal control strain L104. **a** Aligned dot plot of the relative CFU counts of the individual strains. Red: MLVA complex A; green: MLVA complex C; blue: MLVA complex B. The horizontal line indicates the mean. **b** Box plot comparing relative CFU counts between complex A and C strains. CFU counts are significantly higher in brain-slices infected with complex A strains, * = *p* < 0.05. **c** Box plot comparing relative CFU counts between complex A and C strains isolated from ruminant rhombencephalitis. **d** Box plot comparing relative CFU counts according to host species. Whiskers represent maxima and minima. The horizontal line represents the median, + is the mean

**Fig. 2:**
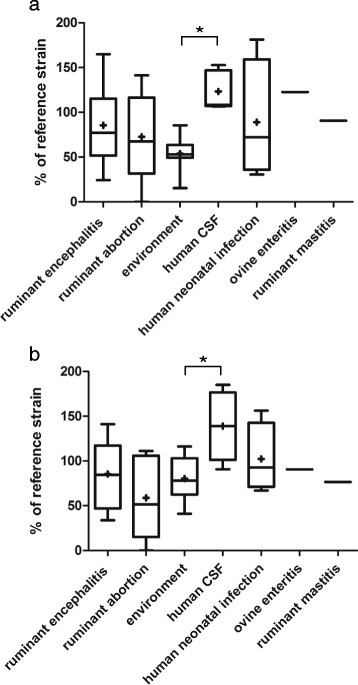
Immunofluorescence stained confocal images of bacteria in infected brain-slices. **a** Delineation of an infection focus (yellow line). *L. monocytogenes* are stained in red. The surface area covered by *L. monocytogenes* was drawn and calculated using the Fluoview software (Olympus FV10-ASW Version 03.01.01.09). Magnification 20×. **b** Representative double-immunofluorescence of a *L. monocytogenes* infected brain-slice. The vast majority of bacteria are found within microglia. Left: Microglia are stained with CD68 in green. Center: *L. monocytogenes* in red. Right: Overlay (bar = 40 μm)

**Fig. 3:**
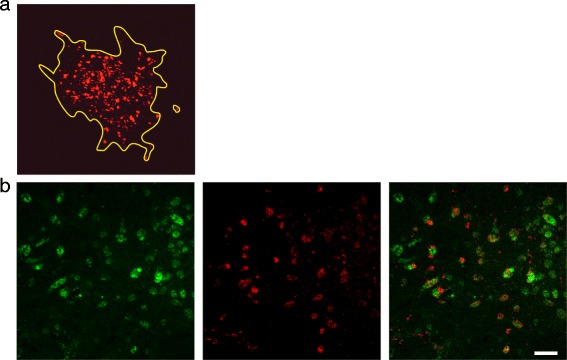
Spread of *L. monocytogenes* strains in brain-slices as determined by size of infection foci. Results are shown relative to the internal control strain L104. **a** Aligned dot plot analysis of bacterial spread of the individual strains used in this study. Red: MLVA complex A; green: MLVA complex C; blue: MLVA complex B. The horizontal line indicates the mean. **b** Box plots comparing the total size of foci between complex A and complex C strains. Complex A strains cover a significantly larger area than complex C strains. **c** Box plot comparing total size of foci according to host species. Human strains caused larger infection foci in brain-slices than strains isolated from small ruminants. Box plots: Whiskers represent maxima and minima. The horizontal line represents the median, + is the mean, * = *p* < 0.05

**Fig. 4:**
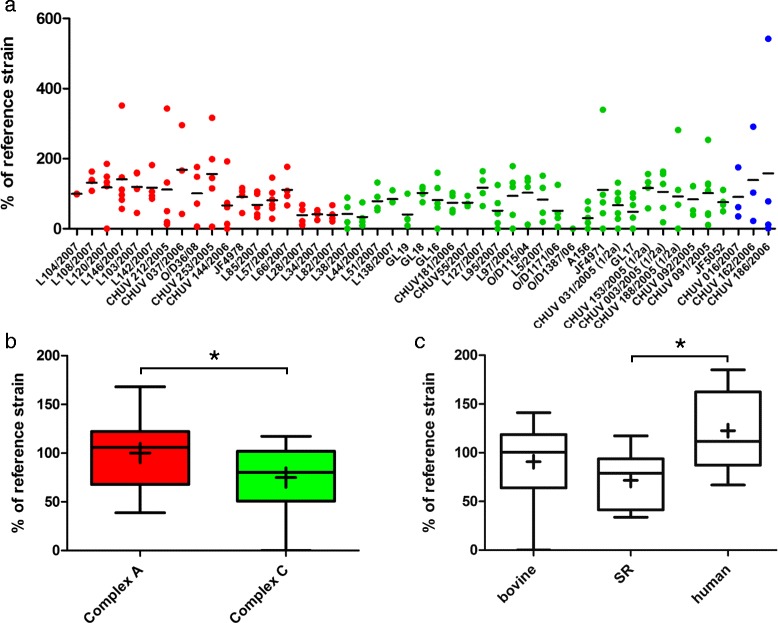
CFU counts (**a**) and size of infection foci (**b**) in organotypic brain-slices infected with *L. monocytogenes* strains. Results are mapped according to the source and associated clinical infection, respectively. Data are presented as box plots, Whiskers represent maxima and minima. The vertical line represents the median, + is the mean. * = *p* < 0.05. CFU counts (**a**) and surface of bacterial spread (**b**) are shown relative to the internal control strain L104

We analyzed replication and capacity to spread in organotypic hippocampal brain-slice cultures of 21 *L. monocytogenes* strains isolated from ruminant rhombencephalitis cases and 26 strains of ruminant non-encephalitic cases (abortion, mastitis, gastroenteritis), human clinical infections or food/environment (Additional file 1). All but one (bovine abortion, O/D1387/06) replicated in the brain slices (Fig. 1a) and established at least one visible focus of infection at 48 h post inoculation (Fig. 2a). Typically, values between 10^5^ and 10^7^ CFU were recovered from the brain-slices at 48 h post infection, which corresponds to a 10^3^–10^5^ times increase over the incubation period. Recovered CFU numbers were significantly higher in brain-slices infected with *L. monocytogenes* strains from MLVA complex A than with strains from MLVA complex C (Fig. 1b, *p* < 0.001). The difference between MLVA complexes was also apparent with encephalitis strains, although as a statistically non-significant tendency (*p* = 0.055, Fig. 1c). Additionally, strains from complex A spread over a significantly larger area than strains from complex C (Fig. 3a and b, *p* = 0.03). No differences in CFU counts were detected when comparing strains by host species or source except between human strains isolated from cerebrospinal fluid (CSF) and environmental strains (Fig. 1d and Fig. 4a). Human CSF strains spread farther than strains isolated from small ruminants (Fig. 3c) and from the environment (Fig. 4b).

In agreement with our previous study [42], immunofluorescence with *L. monocytogenes* antibodies and cell markers revealed that the vast majority of bacteria were located in the cytoplasm of microglia, irrespective of the strain (Fig. 2b). Only a few bacteria were associated with neurofilaments or astrocytes (data not shown).

### Plaque forming assays in cell lines

**Fig. 5:**
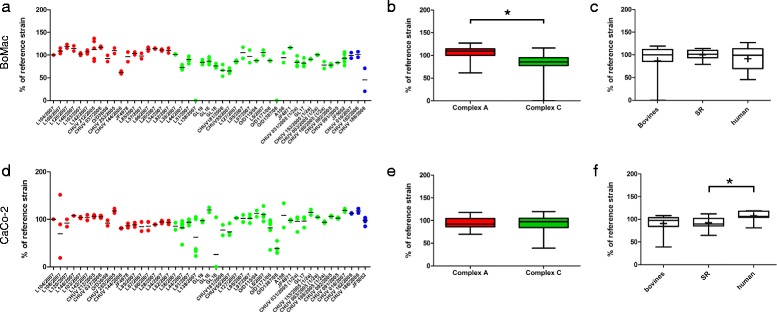
Plaque sizes of *L. monocytogenes* strains in the BoMac and CaCo-2 cell lines. Results are shown relative to the internal control strain L104. **a** The relative plaque size in BoMac-cells is shown for each strain as an aligned dot plot. Red: MLVA complex A strains; green: MLVA complex C strains; blue: MLVA complex B strains. The horizontal line indicates the mean. **b** Box plots comparing plaque size in BoMac cells between complex A and complex C strains. Plaques of complex A strains are significantly larger than those of complex C strains. The horizontal line represents the median, + is the mean. **c** The host species had no influence on plaque-size in BoMac cells. **d** CaCo-2 cells: the relative plaque size for each strain is shown as an aligned dot plot. Red: Complex A; green: Complex C; blue: Complex B. The horizontal line indicates the mean. **e** Box plots comparing plaque size in CaCo-2 cells between complex A and complex C strains. There is no difference in plaque size between complex A and C strains. **e** Human strains formed larger plaques in CaCo-2 cells than strains isolated from small ruminants. * = *p* < 0.05. Box plots: Whiskers represent maxima and minima. The horizontal line represents the median, + is the mean

To assess whether the results obtained in the brain-slice infection assay are tissue specific, we performed plaque forming assays [43,44] in two cell lines: BoMac (bovine macrophages) and CaCo-2 (human colorectal adenocarcinoma cell line). In parallel to the results in brain-slices, all strains except O/D1387/06 caused plaques in the BoMac cell line (Fig. 5a), and strains from MLVA complex A caused significantly larger plaques than strains from complex C (Fig. 5b). In contrast, plaque assays in CaCo-2 cells demonstrated no correlation between plaque size and MLVA-complex (Fig. 5e). Intriguingly, strain O/D1387/06 caused plaques half the size of the reference strain in this cell line, although it made no plaques in BoMac cells (Fig. 5d). Also, the mean absolute plaque size of *L. monocytogenes* strains was significantly larger in CaCo-2 cells than in BoMac cells (1.3 vs 0.9 mm, *p* < 0.001). In the Caco-2 cell line (Fig. 5f), but not in the BoMac cell line (Fig. 5c), human clinical strains produced larger plaques than strains isolated from small ruminants.

## Discussion

Given the ubiquitous presence of *L. monocytogenes* in the environment, discrimination of neurovirulent subtypes would be highly desirable for surveillance purposes and effective disease control, especially with regard to the high mortality rate [35,45] and the high frequency of persistent neurologic deficits associated with neurolisteriosis [10,41]. In our study, infection assays with *L. monocytogenes* strains from various sources and MLVA complexes showed that all *L. monocytogenes* strains except one are able to replicate and spread in bovine brain-slices and that discrimination between rhombencephalitis and non-encephalitic strains is not possible in this system. Hence, based on our results we suggest that all *L. monocytogenes* strains should be regarded as potentially neurovirulent, independent of their genotype and source of isolation. Brain-slices have the inherent drawback that they only model the intracerebral phase of *L. monocytogenes* infection and can not mimic invasion barriers (*i.e.* the blood–brain barrier in hematogenous infection or the cranial nerve in rhombencephalitis). Hence, we cannot exclude that encephalitogenic strains diverge from other *L. monocytogenes* strains due to their efficiency of brain invasion.

MLVA complex A strains (lineage I) showed a higher replication and spread farther in brain-slices than strains from complex C, indicating that they are better adapted to establish encephalitis. This observation is in accordance with the higher prevalence of lineage I strains in clinical infections and in particular ruminant rhombencephalitis compared to lineage II strains [21,22,31]. Analysis of strains according to their source did not identify significant differences in replication and spread in brain-slices except between human and environmental or small ruminant isolates. However, these observations are likely to be related to the MLVA complex of the strains. All human CSF strains belonged to MLVA complexes A and B (lineage I), whereas all environmental and the majority of small ruminant strains belonged to complex C (lineage II). Microglia, the innate immune cells and resident macrophages of the CNS [46], were the main target cells for all *L. monocytogenes* strains investigated in hippocampal brain slices. The replication of *L. monocytogenes* within microglia indicates that these may paradoxically act as a replication niche for *L. monocytogenes* during encephalitis. In this aspect, our model is consistent with the natural disease, where most bacteria are found within phagocytes of microabscesses [11].

MLVA complex A strains form larger plaques in the bovine macrophage cell line (BoMac) than complex C strains. Interestingly, this difference was not apparent in CaCo-2 cells, where plaques were generally larger than in BoMac cells, indicating a particularly high susceptibility of epithelial cells to *L. monocytogenes,* which does not allow further differentiation. This notion is further supported by the fact that strain O/D1387/06, a complex C strain that seems to be naturally attenuated, completely failed to replicate and spread in brain-slices and to cause plaques in BoMac cells, but caused small plaques in CaCo-2 cells. Further analysis of this strain revealed a novel PrfA truncation, associated with the attenuated phenotype *in vitro* [47].

Our data suggests that differences in replication and spread between *L. monocytogenes* strains are host cell type-specific. Contradictory experimental data on *L. monocytogenes* virulence from studies using various types of cell lines may support this view [44,48–54]. Unlike CaCo-2 cells, BoMac (derived from bovine macrophages) and microglia share essential features including phagocytic potential and a respiratory burst system [46]. Taken together, the more efficient replication and spread of MLVA complex A vs. complex C strains in microglia and macrophages suggests that complex A strains are more resistant to mononuclear antibacterial defense mechanisms. In this context, it should be noted that listeriolysin S, a virulence factor induced by oxidative stress, has been implicated in *L. monocytogenes* survival within phagocytes and is specifically expressed by strains of lineage I [55].

## Conclusion

Our data demonstrates that all *L. monocytogenes* strains should be considered potentially neurovirulent and encephalitis strains cannot be conclusively discriminated from non-encephalitis strains using the bovine organotypic brain-slice model. In this CNS model, microglia cells are the main target cells for all tested *L. monocytogenes* strains, which are able to multiply in these phagocytic cells. Correlation of MLVA data with our *in vitro* data show that strains from MLVA complex A replicate and spread better in bovine microglia and macrophages possibly by virtue of their higher resistance to mononuclear antibacterial defense mechanisms. These results support the notion that *L. monocytogenes* strains from MLVA complex A are highly accomplished at establishing encephalitis.

## Methods

### Bacterial strains

Forty-seven *L. monocytogenes* strains were investigated in brain-slice cultures and cell lines (Additional file 1). The MLVA-type of 44 strains had been obtained in a previous study [31] and the MLVA-type of three other ruminant isolates (JF4971, JF5052 and JF4978; Additional file 1) were determined during this study by analysis of tandem repeat numbers at eight loci according to Sperry *et al.* [23]. A minimal spanning tree was created in the BioNumerics software (Version 6.6, Applied Maths Inc., Austin, Texas, USA) in order to define the MLVA complex of the strains [31]. Twenty-one strains isolated from ruminant rhombencephalitis cases were selected based on the following criteria: 1) differences at the 8 MLVA loci and 2) similar numerical representation of the two large MLVA complexes, to which most of the ruminant rhombencephalitis strains belong (MLVA complex A: *n* = 12; MLVA complex C: *n* = 9). The ruminant rhombencephalitis strains were compared to a similar number of *L. monocytogenes* strains from other sources (*n* = 26) available in our strain collection (Additional file 1). The latter included strains from ruminant non-encephalitic cases including gastroenteritis, mastitis and abortion (*n* = 7), human clinical cases (*n* = 9), food and environmental (*n* = 10) and mainly belonged to MLVA complex C (*n* = 18). Four strains belonged to MLVA complex A, one strain was a single locus variant associated with MLVA complex A and three strains belonged to MLVA complex B. Non-invasive *Listeria innocua* type strain CCUG15531^T^ (Culture Collection University of Göteborg) was used as negative control.

### Organotypic brain-slice cultures

Hippocampal brain samples from calves under 6 months of age were obtained from the slaughterhouse. A vibratome (Leica VT1000S) was used to cut 350 μm brain-slices and were cultured on membrane inserts (Vitaris, No. 3450 or 3460) as previously described [42].

### Infection assays in ruminant organotypic brain-slice cultures

Brain-slices were infected at day 7 in culture. Penicillin and streptomycin were removed from the organotypic brain-slice cultures 1 h prior to inoculation in the first set of experiments and 4 days prior to inoculation in the later experiments due to batch variation of the antibiotics. Medium was changed to a serum-free formula 1 h prior to inoculation. Bacteria were plated on trypticase soy agar (TSA), incubated at 37 °C for 15 h and subsequently diluted using the McFarland optical density standard. One hundred CFU in 0.1 μl NaCl (determined by plating on TSA plates) were focally injected into the dentate gyrus of the hippocampus using a 0.5 μl syringe (Hamilton, 7402 Bonaduz, Switzerland. Model 7000.5 KHOC) and a micromanipulator (made in-house). Brain-slices were incubated with the bacteria for 3 h and subsequently the inoculation medium was substituted with gentamicin-containing medium (final concentration 0.01 mg/ml). All experiments were carried out at least in triplicate and included *L. innocua* (negative control) and an internal control strain (L104, from bovine rhombencephalitis, MLVA complex A) for normalization. For analysis of bacterial replication CFU’s were determined 48 h post infection by lysing infected brain-slices in 1 ml PBS containing 55 μl Isolator (Wampole, Oxoid) and plating serial dilutions on TSA plates. For analysis of bacterial spread, brain-slices were fixed in 4 % (w/v) paraformaldehyde at 48 h post infection. Following overnight fixation, brain-slices were incubated in 18 % (w/v) sucrose (Sigma, S0389) for 12 h, cut with a cryotome into 4.5 μm-thick sections and stored at −20 °C until further use. Immunofluorescence was performed using the following primary antibodies: anti-Listeria O serotypes 1 and 4 (polyclonal rabbit antibody, No. 223021, Difco, Sparks, MD, USA), neurofilament (monoclonal mouse antibody, No M0762, DAKO, Glostrup, Denmark), GFAP (monoclonal mouse antibody, No. Ab4648, Abcam, Cambridge UK) and CD68 (monoclonal mouse antibody, clone EBM11, DAKO, Glostrup, Denmark). Alexa Fluor 488 and 544 were used as secondary antibodies (No. A21428, Invitrogen, Carlsbad, CA, USA,) [42]. Nuclei were stained with TOTO-3 (no T3604, Invitrogen, Carlsbad, CA USA), and 10x images were acquired on an Olympus FV1000 confocal microscope. The total area covered by *L. monocytogenes* was measured on the immunofluorescence labeled cryosections of brain-slices using the Olympus FV10-ASW Version 03.01.01.09 software and expressed in μm^2^.

### Plaque assay in bovine macrophages and CaCo-2 cells

Plaque forming assays were performed according to a previous study [31] in an immortalized bovine macrophage cell line (BoMac, kindly provided by D. Dobbelaere, Department of Clinical Research and Veterinary Public Health, Vetsuisse Faculty Bern) and the human enterocyte-like CaCo-2 cell line (ATCC No. HTB37). Both cell lines were grown in DMEM (Gibco 61965–026) supplemented with penicillin/streptomycin (Gibco, 15140–122, used 1:100) and 10 % fetal calf serum (FCS) (BoMac cells) or 20 % FCS (CaCo-2 cells), respectively. Cells were grown to confluence in a 24-well plate overnight at 37 °C, washed with warm PBS and inoculated with 10^3^ CFU *L. monocytogenes* (MOI 0.01) in antibiotic free medium supplemented with 2 % FCS. Following 1 h incubation the medium was removed, cells were washed with PBS and overlaid with medium containing 0.7 % agarose and 0.01 mg/ml gentamicin. The size of five randomly chosen plaques per well were measured 72 h post infection. Experiments were carried out in duplicate and the internal control strain L104 was included in all experiments.

### Statistical analysis

All results were normalized to the internal control strain L104. Statistical analysis was performed with the Prism Software (Version 5.03, Graph Pad Software Inc.). The Mann–Whitney test was used to determine the p-values where two groups were compared. For comparison of multiple groups, the Kruskal-Wallis test was used with Dunn’s multiple comparisons as post-test. As only three strains belonged to complex B, they were excluded from the statistical analysis comparing complexes.

## Additional file

Additional file 1:
***L. monocytogenes*** **strains used in this study. MLVA and serotype data have been either obtained from** [31] **or were generated in this study (*).** *SLV* Single locus variant, *N/a* Not applicable, *Nd* Not determined, *CSF* Cerebrospinal fluid.
