# Erratum: Increased spread and replication efficiency of *Listeria monocytogenes* in organotypic brain-slices is related to multilocus variable number of tandem repeat analysis (MLVA) complex

**DOI:** 10.1186/s12866-015-0492-7

**Published:** 2015-09-03

**Authors:** Claudia Guldimann, Michelle Bärtschi, Joachim Frey, Andreas Zurbriggen, Torsten Seuberlich, Anna Oevermann

**Affiliations:** Division of Neurological Sciences, Neurocenter, Department of Clinical Research and Veterinary Public Health, Vetsuisse Faculty, University of Bern, Bern, Switzerland; Graduate school for Cellular and Biomedical Sciences, University of Bern, Bern, Switzerland; Institute of Veterinary Bacteriology, Vetsuisse Faculty, University of Bern, Bern, Switzerland

## Erratum

The original version of this article unfortunately contained a mistake. Figures two, three and four (Figs. 1, 2 and 3 here, respectively) and their associated legends were interchanged in the HTML and PDF versions of this manuscript. The correct versions are given below. In addition, Figure Five (Fig. 4 here) was missing in the HTML version of this manuscript. The correct figure Five (Fig. 4 here) is also included below.

**Fig. 1:**
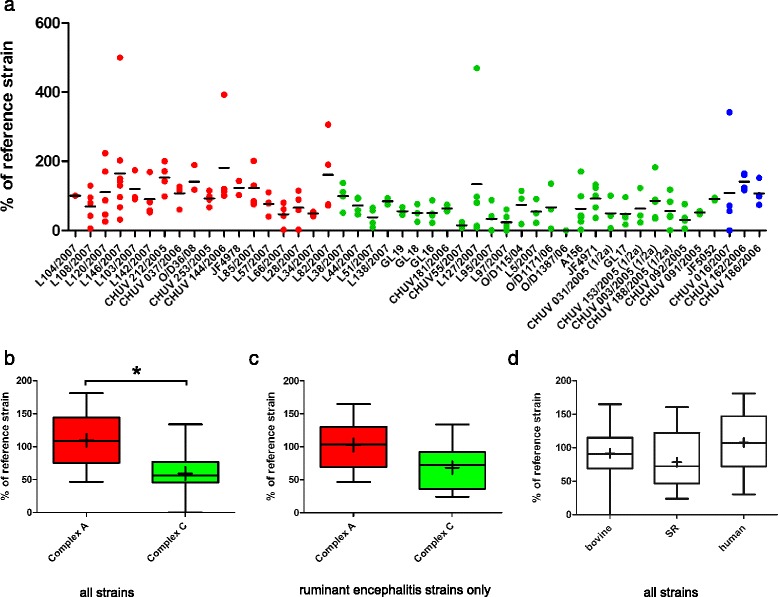
Immunofluorescence stained confocal images of bacteria in infected brain-slices. **a**: Delineation of an infection focus (yellow line). L. monocytogenes are stained in red. The surface area covered by L. monocytogenes was drawn and calculated using the Fluoview software (Olympus FV10-ASW Version 03.01.01.09) Magnification 20x. **b**: Representative double-immunofluorescence of a L. monocytogenes infected brain-slice. The vast majority of bacteria are found within microglia. Left: Microglia are stained with CD68 in green. Center: L. monocytogenes in red. Right: Overlay (bar = 40 μm)

**Fig. 2:**
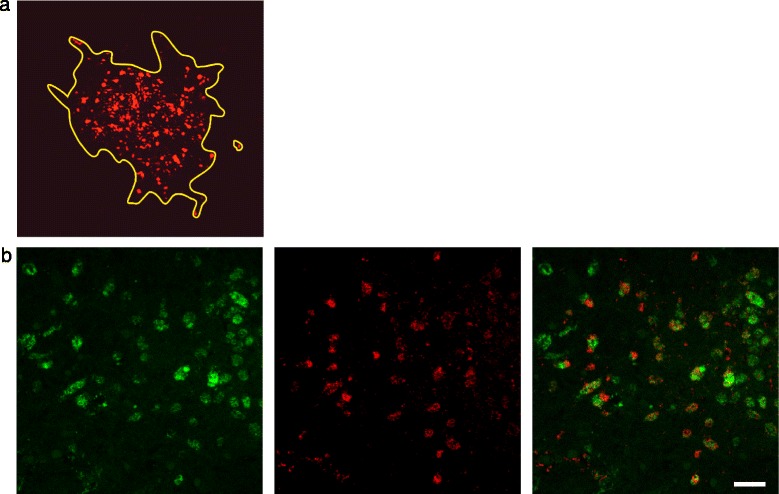
Spread of L. monocytogenes strains in brain-slices as determined by size of infection foci. Results are shown relative to the internal control strain L104. **a**: Aligned dot plot analysis of bacterial spread of the individual strains used in this study. Red: MLVA complex A; green: MLVA complex C; blue: MLVA complex B. The horizontal line indicates the mean. **b**: Box plots comparing the total size of foci between complex A and complex C strains. Complex A strains cover a significantly larger area than complex C strains. **c**: Box plot comparing total size of foci according to host species. Human strains caused larger infection foci in brain-slices than strains isolated from small ruminants. Box plotss: Whiskers represent maxima and minima. The horizontal line represents the median, + is the mean, * = *p* < 0.05

**Fig. 3:**
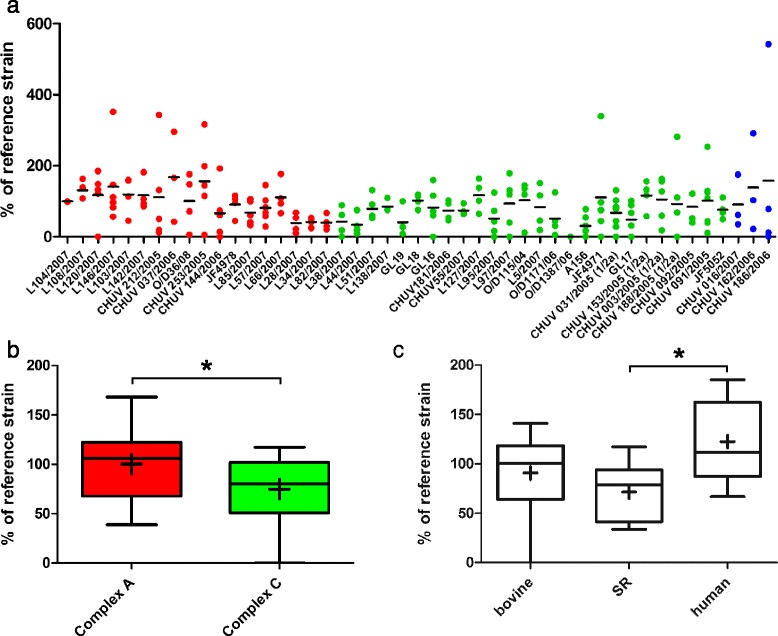
CFU counts (**a**) and size of infection foci (**b**) in organotypic brain-slices infected with L. monocytogenes strains. Results are mapped according to the source and associated clinical infection, respectively. Data are presented as box plots, Whiskers represent maxima and minima. The vertical line represents the median, + is the mean. * = *p* < 0.05. CFU counts (**a**) and surface of bacterial spread (**b**) are shown relative to the internal control strain L104

**Fig. 4:**
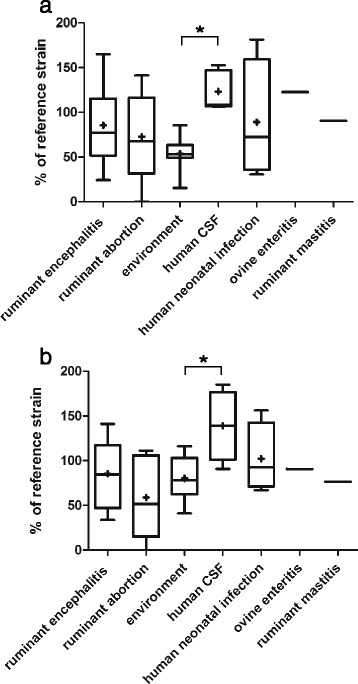
Plaque sizes of *L. monocytogenes* strains in the BoMac and CaCo-2 cell lines. Results are shown relative to the internal control strain L104. **a**: The relative plaque size in BoMac-cells is shown for each strain as an aligned dot plot. Red: Complex A strains; green: Complex C strains; blue: Complex B strains. The horizontal line indicates the mean. **b**: Box plots comparing plaque size in BoMac cells between complex A and complex C strains. Plaques of complex A strains are significantly larger than those of complex C strains. The horizontal line represents the median, + is the mean. **c**: The host species had no influence on plaque-size in BoMac cells. **d**: CaCo-2 cells: the relative plaque size for each strain is shown as an aligned dot plot. Red: Complex A; green: Complex C; blue: Complex B. The horizontal line indicates the mean. **e**: Box plots comparing plaque size in CaCo-2 cells between complex A and complex C strains. There is no difference in plaque size between complex A and C strains. **f**: Human strains formed larger plaques in CaCo-2 cells than strains isolated from small ruminants. * = *p* < 0.05. Box plots: Whiskers represent maxima and minima. The horizontal line represents the median, + is the mean

