# Supplementary material for: Increased spread and replication efficiency of Listeria monocytogenes in organotypic brain-slices is related to multilocus variable number of tandem repeat analysis (MLVA) complex
Source: BMC Microbiol. 2015 Jul 3;15:134. doi: 10.1186/s12866-015-0454-0 (PMC4490720; doi:10.1186/s12866-015-0454-0)
Supplement: Additional file 1: — L. monocytogenes strains used in this study. MLVA and serotype data have been either obtained from [31] or were generated in this study (*). SLV Single locus variant, N/a Not applicable, Nd Not determined, CSF Cerebrospinal fluid. [file 12866_2015_454_MOESM1_ESM.pdf]

Strain designation	strain information				PCR serovar	MLVA complex	MLVA locus copy number							
	source	host species	Country	Year			Lm-2	Lm-8	Lm-10	Lm-11	Lm-3	Lm-23	Lm-15	Lm-32
L108/2007	brain (rhombencephalitis)	bovine	CH	2007	4b; 4d; 4e	A	16	3	2	4	1	16	2	18
L120/2007	brain (rhombencephalitis)	bovine	CH	2007	4b; 4d; 4e	A	16	3	2	4	1	17	2	18
L104/2007	brain (rhombencephalitis)	bovine	CH	2007	4b; 4d; 4e	A	16	3	2	4	2	17	2	18
L146/2007	brain (rhombencephalitis)	bovine	CH	2007	4b; 4d; 4e	A	17	3	2	4	1	17	2	18
L103/2007	brain (rhombencephalitis)	bovine	CH	2007	4b; 4d; 4e	A	17	3	2	4	1	22	2	18
L142/2007	brain (rhombencephalitis)	bovine	CH	2007	4b; 4d; 4e	A	17	3	2	4	2	17	2	18
L57/2007	brain (rhombencephalitis)	sheep	CH	2007	4b; 4d; 4e	A	16	3	2	4	1	17	1	18
L28/2007	brain (rhombencephalitis)	sheep	CH	2007	4b; 4d; 4e	A	17	3	2	4	1	17	2	18
L82/2007	brain (rhombencephalitis)	sheep	CH	2007	4b; 4d; 4e	A	17	3	2	4	2	16	2	18
L85/2007	brain (rhombencephalitis)	sheep	CH	2007	4b; 4d; 4e	A	17	3	2	4	2	17	2	18
L66/2007	brain (rhombencephalitis)	sheep	CH	2007	4b; 4d; 4e	A	17	3	2	5	2	17	2	18
L34/2007	brain (rhombencephalitis)	sheep	CH	2007	4b; 4d; 4e	A	22	3	2	4	2	17	2	18
L138/2007	brain (rhombencephalitis)	bovine	CH	2007	1/2a; 3a	C	18	2	2	4	7	20	1	14
L127/2007	brain (rhombencephalitis)	goat	CH	2007	1/2a; 3a	C	16	2	2	3	4	20	3	14
L95/2007	brain (rhombencephalitis)	goat	CH	2007	1/2a; 3a	C	17	2	2	4	10	14	1	13
L51/2007	brain (rhombencephalitis)	sheep	CH	2007	1/2a; 3a	C	16	2	2	3	1	21	1	13
L38/2007	brain (rhombencephalitis)	sheep	CH	2007	1/2b; 3b; 7	C	16	2	2	3	4	18	3	14
L44/2007	brain (rhombencephalitis)	sheep	CH	2007	1/2a; 3a	C	18	2	2	3	1	18	1	14
L97/2007	brain (rhombencephalitis)	sheep	CH	2007	1/2a; 3a	C	18	2	2	3	6	22	1	13
O/D115/04	brain (rhombencephalitis)	sheep	CH	2004	nd	C	19	2	2	3	10	20	2	14
L5/2007	brain (rhombencephalitis)	sheep	CH	2007	1/2a; 3a	C	19	2	2	4	9	20	2	14
CHUV 212/2005 (4b)	CSF	human	CH	2005	4b; 4d; 4e	A	17	3	2	4	2	17	3	19
CHUV 037/2006 (4b)	CSF	human	CH	2006	nd	A	17	4	2	4	1	17	2	18
CHUV 016/2007 (4b)	CSF	human	CH	2007	nd	B	16	2	4	3	2	14	4	17
CHUV 162/2006 (4b)	CSF	human	CH	2006	4b; 4d; 4e	B	16	2	4	3	2	14	4	17
CHUV 186/2006 (4b)	CSF	human	CH	2006	4b; 4d; 4e	B	16	2	4	3	3	14	4	17
JF5052	udder (mastitis)	bovine	CH	2010	nd	C *	15	2	3	0	10	40	1	13
JF4971	placenta (abortion)	bovine	GB	2004	nd	C *	16	2	5	4	6	22	2	13
O/D36/08	placenta (abortion)	bovine	CH	2008	4b; 4d; 4e	C	17	3	2	4	2	17	3	18
O/D1387/06	placenta (abortion)	bovine	CH	2006	1/2a; 3a	C	19	2	2	3	1	14	2	14
A156	placenta (abortion)	bovine	CH	2010	nd	C	19	2	2	3	8	21	2	14
O/D1171/06	placenta (abortion)	bovine	CH	2006	1/2a; 3a	C	21	2	2	3	2	10	6	15
JF4978	abomasal content (gastroenteritis)	sheep	GB	2004	nd	SLV associated with A *	15	3	5	5	5	17	3	16
CHUV 144/2006 (4b)	neonatal infection	human	CH	2006	4b; 4d; 4e	A	5	3	2	4	1	17	2	18
CHUV 253/2005 (4b)	neonatal infection	human	CH	2005	4b; 4d; 4e	A	17	3	2	5	2	17	2	18
CHUV 091/2005 (1/2a)	neonatal infection	human	CH	2005	nd	C	18	2	2	3	7	19	1	14
CHUV 092/2005 (1/2a)	neonatal infection	human	CH	2005	nd	C	19	2	2	3	7	19	1	14
CHUV55/2007 (1/2a)	food	n/a	CH	2007	nd	C	21	2	2	3	2	11	1	14
CHUV181/2006 (1/2a)	food	n/a	CH	2006	1/2c; 3c	C	22	2	2	3	7	11	1	14
CHUV 188/2005 (1/2a)	environment	n/a	CH	2005	nd	C	15	2	2	3	8	19	2	14
CHUV 031/2005 (1/2a)	environment	n/a	CH	2005	nd	C	17	2	2	3	1	20	3	14
CHUV 153/2005 (1/2a)	environment	n/a	CH	2005	1/2a; 3a	C	18	2	2	4	6	22	1	13
CHUV 003/2005 (1/2a)	environment	n/a	CH	2005	nd	C	19	2	2	3	5	23	1	14
GL18	cheese	n/a	GR	2006	1/2c; 3c	C	18	2	2	3	1	3	1	13
GL16	cheese	n/a	GR	2006	1/2c; 3c	C	18	2	2	3	5	7	1	13
GL19	cheese	n/a	GR	2006	1/2a; 3a	C	19	2	2	2	5	8	1	14
GL17	cheese	n/a	GR	2006	1/2c; 3c	C	21	2	2	3	6	1	1	13
